# Improvement in the Extraction of Antioxidant-Related Compounds from *Parastrephia quadrangularis* (“tola”) Using Ethanol-Modified Supercritical Carbon Dioxide

**DOI:** 10.3390/antiox15030303

**Published:** 2026-02-28

**Authors:** Paula Ardiles, Francisca Salinas-Fuentes, July Z. Florez, Juan Luis Fuentes, Daniel Ordenes, Waldo Bugueño, Jenifer Palma, María Robles, María Cuaresma, Carlos Vilchez, Pedro Cerezal-Mezquita, Mari Carmen Ruiz-Domínguez

**Affiliations:** 1 Laboratory of Microencapsulation of Bioactive Compounds (LAMICBA), Department of Food Science and Nutrition, Faculty of Health Sciences, University of Antofagasta, Antofagasta 1240000, Chile; paula.ardiles.alvarez@ua.cl (P.A.); francisca.salinas@uantof.cl (F.S.-F.); waldo.bugueno@uantof.cl (W.B.); jenifer.palma@uantof.cl (J.P.); 2 Centro i-mar, CeBiB & MASH, Universidad de Los Lagos, Puerto Montt 5480000, Chile; 3 Extremophile Laboratory, Centro de Investigación en Química Sostenible (CIQSO), I3B and CIDERTA, University of Huelva, 21007 Huelva, Spain; jlfuentes@dqcm.uhu.es (J.L.F.); daniel.ordenes@alu.uhu.es (D.O.); maria.robles@dqcm.uhu.es (M.R.); maria.cuaresma@dqcm.uhu.es (M.C.); cvilchez@uhu.es (C.V.); 4 Atacama BioNatural Products S.A. Company, Iquique 1100000, Chile; pcerezal@atacamabionatural.com

**Keywords:** antioxidant activity, Asteraceae, fatty acids, gas chromatography–mass spectrometry, green extraction, molecular identification, phenolic compounds, supercritical fluid extraction

## Abstract

*Parastrephia quadrangularis* (tola) is a native plant of the Chilean Andean Altiplano that is traditionally used for its anti-inflammatory properties. In this study, the aerial parts of the plant were analysed to determine their fatty acid (FA) profile and to identify bioactive compounds using gas chromatography–mass spectrometry (GC–MS). Both conventional extraction methods and Supercritical Fluid Extraction (SFE) were employed, using a 2^3^ factorial design with centre-point replicates. The variables included temperature (30–60 °C), pressure (15–45 MPa), and ethanol as a cosolvent (0–30% *v*/*v*). Extraction kinetics were evaluated using a linear spline model under central conditions (45 °C, 30 MPa, 15% ethanol). Response variables included extraction yield, Total Phenolic Content (TPC), antioxidant activity measured by Trolox Equivalent Antioxidant Capacity (TEAC), and FA composition. A factorial design identified pressure and ethanol concentration as key drivers of phenolic content and antioxidant activity, as supported by confocal autofluorescence microscopy. Multi-response optimisation based on the desirability function was applied to simultaneously maximise all response variables, yielding predicted optimal extraction conditions at 60 °C, 45 MPa, and 30% *v*/*v* ethanol for *P. quadrangularis*. The FA profile highlighted polyunsaturated FAs such as oleic, linoleic, and linolenic acids, as well as saturated FAs including palmitic and lignoceric acids, and short-chain non-volatile FAs. GC–MS analysis revealed metabolites potentially responsible for the plant’s traditionally reported therapeutic effects. Overall, these results highlight ethanol-based SFE as a sustainable strategy for recovering phenolic compounds and antioxidant-related fractions from ancestral medicinal plants.

## 1. Introduction

Plant-derived chemical compounds have attracted increasing interest from the pharmaceutical and food industries due to their broad potential for biological and technological applications [1]. Several studies have identified bioactive compounds in herbaceous plants used in traditional folk medicine [2].

In the northern Chilean region of Arica y Parinacota and Antofagasta, native plants are commonly used by the Quechua and Aymara cultures in traditional practices that have been passed down through generations. Many plants in this area belong to the Asteraceae, one of the most diverse families of herbaceous plants in the Chilean flora. Among them are members of the genus *Parastrephia*, which includes four major species: *P. lucida*, *P. lephidophylla*, *P. quadrangularis*, and *P. teretiuscula*, all commonly known as “tola” [3]. This herb is usually found in the Puna belt, located at high altitude, between 3200 and 4200 m above sea level. It is characterised by high, constant ultraviolet radiation, low oxygen levels, extreme thermal oscillations during the day, and significant water shortage (<400 mm of water per year) [3,4].

*Parastrephia* spp. has traditionally been used to treat inflammation associated with various conditions, including body pain like headaches, altitude sickness, fevers, and toothaches, as well as urinary, gastric, and respiratory infections, fractures, contusions, and wound healing [5]. Previous studies have reported the presence of phenolic compounds such as caffeic acid, ferulic acid, quercetin, and kaempferol, which are associated with antioxidant, anti-inflammatory, antimicrobial, and anticancer activities [6,7,8]. The *Parastrephia* genus has so far been hardly explored for other compounds of interest, a pursuit that is of interest given the increasing interest in natural sources of phenolic compounds as alternatives to synthetic agents [2]. In this study, the species genus was confirmed using molecular biology techniques. The species were explored for the presence of other bioactive compounds extracted using green methods, such as the fatty acid profile, total phenols’ presence, antioxidant activity analysed by antioxidant activity measured by the Trolox Equivalent Antioxidant Capacity (TEAC), and other natural molecules with relevant properties determined by gas chromatography–mass spectrometry (GC–MS).

Despite its long-standing traditional use and adaptation to the extreme environmental conditions of the Andean Altiplano, *Parastrephia quadrangularis* remains insufficiently characterised with respect to how sustainable extraction strategies influence the recovery of its bioactive fractions. Most previous studies have focused on conventional solvent-based extractions or specific phenolic compounds, leaving a knowledge gap regarding the modulation of polar and non-polar antioxidant-related fractions using green technologies. Accordingly, the working hypothesis of this study is that ethanol-modified supercritical CO_2_ extraction can selectively tailor the chemical composition and antioxidant potential of *P. quadrangularis* extracts, enabling the recovery of a broader spectrum of bioactive compounds. In this context, in line with the United Nations’ 2030 Agenda for Sustainable Development (https://sdgs.un.org/goals, accessed on 9 January 2026), green extraction technologies such as Supercritical Fluid Extraction (SFE) have emerged as promising alternatives to conventional solvent-based methods, which are often time and solvent-intensive and may compromise product sustainability [9,10,11]. Moreover, the application of response surface methodology (RSM) provides an efficient statistical framework to evaluate the interaction between extraction parameters, reduce experimental effort, and identify favorable operating conditions for the recovery of bioactive compounds.

Therefore, this study aims to evaluate and improve conventional solvent extraction and green SFE for the recovery of bioactive compounds from the plant molecularly identified as *P. quadrangularis*, a traditional Andean species with potential therapeutic value. Ethanol, selected as the most effective green solvent in conventional extraction, was subsequently employed as a cosolvent in SFE. A full factorial experimental design (2^3^) with replicated centre points was applied, followed by desirability-based multiple response optimisation to simultaneously maximise extraction yield, total phenolic content, and antioxidant activity. Extraction kinetics, evaluated using a linear spline model, were further investigated under centre-point conditions, and complementary analytical techniques, including confocal microscopy and mass spectrometry, were employed to characterise bioactive molecules in the extracts, including fatty acids. Finally, this comprehensive study provides additional insight into the presence of bioactive molecules in P. quadrangularis, contributing to the scientific understanding of medicinal plants traditionally used in Andean culture.

## 2. Materials and Methods

### 2.1. Chemicals and Samples

**Figure 1:**
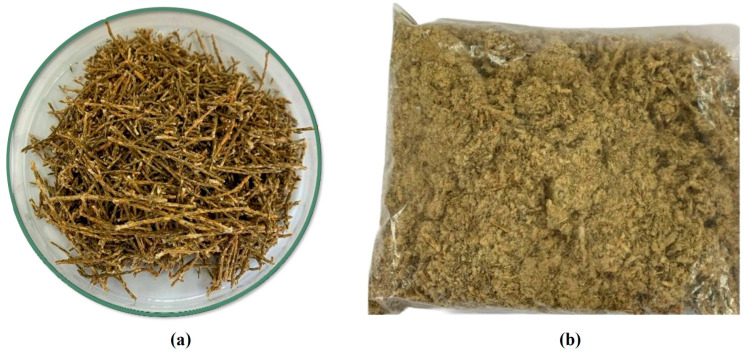
(**a**) Samples of the plant tola were collected from San Pedro de Atacama (Antofagasta, Chile), and (**b**) the plant after milling.

The folk medicinal plant *P. quadrangularis* (commonly known as “tola” or “sipu tola”) was selected for this study. Plant material was purchased from a local market in San Pedro de Atacama (22°55′0″ S; 68°12′0″ W), located at approximately 2400 m above sea level in the Atacama Desert (Antofagasta Region, Chile). To account for seasonal variability, commercial batches were acquired at different times of year, including summer and winter, and subsequently pooled and homogenised prior to processing. The biomass used consisted of aerial parts, including flowers, stems, and leaves (Figure 1), the processing of which is subsequently described. First, the plants were freeze-dried at −48 ± 5 °C under vacuum for 36 h at an absolute pressure of 70–80 Pa using a freeze-drying system (Labconco FreeZone 2.5 L Benchtop Dry System; Labconco, Kansas City, MO, USA). Then, the samples were milled to a fine powder (0.23 ± 0.005 mm) using an IKA A11 basic mill (Ika-Werke, Staufen, Germany), packed in vacuum-sealing plastic bags, and finally stored at room temperature (20 ± 2 °C) in the dark until use. This processed biomass is hereafter referred to as milled tola biomass (MTB).

The chemicals used for SFE were carbon dioxide (99% purity) purchased from Indura Group Air Products (Santiago, Chile) and ethanol (99.5%) purchased from Merck (Darmstadt, Germany). Other chemicals, such as ultrapure water, gallic acid, 6-hydroxy-2,5,7,8-tetramethylchroman-2-carboxylic acid (Trolox, ≥97%), 2,2-azino-bis (3-ethylbenzothiazoline-6-sulfonic acid) (ABTS ≥ 99%), and Folin–Ciocalteu phenol reagent, were purchased from Sigma-Aldrich (Santiago, Chile). For the identification and quantification of fatty acids, a standard fatty acid methyl ester (FAME) mix, C4–C24, supplied by Supelco Analytical (Bellefonte, PA, USA), was used, and tripentadecanoin > 99% (Nu-Check Pre, Inc., Elysian, MN, USA) was used as the internal standard.

### 2.2. DNA Extraction, PCR Amplification, Sequencing and Identification

For plant identification, genomic DNA was extracted independently in two laboratories using the DNeasy PowerSoil Pro Kit (Qiagen, Hilden, Germany), to ensure reproducibility of the molecular identification. The nuclear ribosomal ITS region was amplified using two primer pairs targeting partially overlapping regions (ITS1/ITS2 and ITS5a/ITS4) to maximise amplification success and confirm sequence consistency among closely related *Parastrephia* species. Primer set 1: forward primer ITS1 (5ʹ-TCCGTAGGTGAACCTGCGG-3ʹ) [12], and reverse primer ITS2 (5ʹ-GCTGCGTTCTTCATCGATGC-3ʹ) [13]. Primer set 2: forward primer ITS5a (5ʹ-CCTTATCATTTAGAGGAAGGAG-3ʹ) [14] and reverse primer ITS4 (5ʹ-TCCTCCGCTTATTGATATGC-3ʹ) [12].

The amplification process was the same for both protocols and consisted of an initial denaturation step at 95 °C for 5 min, followed by 30 cycles of denaturation at 95 °C for 15 s, primer annealing at 57 °C for 15 s, and extension at 60 °C for 15 s. A final extension step at 60 °C for 5 min concluded the process. The PCR product was purified using the E.Z.N.A. Cycle Pure kit (Omega Bio-tek, Norcross, GA, USA). Genetic sequencing was outsourced to external service providers (Protocol 1: Secugen S.L., Madrid, Spain; Protocol 2: Austral-omics, Puerto Montt, Chile). Subsequently, the obtained sequences were compared against the National Center for Biotechnology Information (NCBI) database (http://www.ncbi.nlm.nih.gov/) to evaluate homology with previously deposited organisms. The comparison involved assessing the percentage of similarity and alignment between paired sequences.

### 2.3. Phylogenetic Analyses

Phylogenetic analyses were conducted by aligning the study sequence from the publicly available NCBI database using ClustalX (Version 2.1) [15] and the Molecular Evolutionary Genetics Analysis (MEGA) software (Version 11) [16]. Nucleotide alignments were subjected to phylogenetic inference using three methods: maximum likelihood (ML) [17], maximum parsimony (MP) [18], and Neighbor–Joining (NJ) [19]. The phylogenetic trees were reconstructed employing the Maximum-Likelihood (ML) algorithm [20] implemented in Mega 11, with the Tamura-Nei model [16], and the robustness of the trees was assessed using 1000 bootstrap replicates.

### 2.4. Conventional Extraction Method

Two green solvents with different polarities (i.e., water and ethanol) were used for solvent extraction (in triplicate) to determine the best extractant for yield, total phenol content (TPC), and antioxidant activity (TEAC) of MTB. Briefly, 200 mg of lyophilised herbaceous and ground samples were mixed with 10 mL of each solvent containing 0.1% (*w*/*v*) BHT in 15 mL Falcon tubes. The mixtures were shaken for 24 h on a thermo-shaker (DBSC-001 model, MRC Ltd., Holon, Israel) at 30 °C and 250 rpm in the dark. After extraction, the exhausted substrate was centrifuged (Eppendorf 5702, Eppendorf, Germany) at 3000× *g* for 10 min. The precipitate was removed, and the supernatants (extracts) were collected. The solvent was removed by passing a stream of N_2_ over the sample (Flexivap Workstation, model 109A YH-1, Glas-Col, Terre Haute, IN, USA) or by freeze-drying (aqueous phase). Then, all extracts were stored at −20 ± 2 °C until measurements were taken.

### 2.5. Supercritical Fluid Extraction

The samples were extracted using a Speed Helix supercritical extractor (Applied Separation, Allentown, PA, USA), following a method described by Salinas et al. [21]. For each extraction assay, 2.0 g of MTB was weighed out, filled with an appropriate amount of glass beads (Ø = 3 mm), and placed in a 24 mL extraction vessel. Carbon dioxide (CO_2_) was introduced into the system until the target pressure was achieved, after which the setup was held under static conditions for 10 min. The extraction process continued for a total of 40 min, exceeding the Falling Extraction Rate (FER) time, which was applied to optimise the yield from herbaceous samples using the midpoint conditions of the SFE procedure.

The CO_2_ flow rate was monitored using a digital flow meter positioned at the outlet of the sample collection container. Once the static period had ended, the bed’s outlet valve was opened, and the micrometric valve was fine-tuned to maintain a CO_2_ flow of 3.62 g/min. Extracts were collected in vials, and any residual solvent was removed using a gentle nitrogen stream to accurately assess the extraction yield. The dried extracts were then stored in the dark at –20 ± 2 °C until further analysis.

### 2.6. Full Factorial Experimental Design and Desirability Function

As shown in Table 1, a full factorial experimental design (2^3^) with two centre–point runs was used in this study, generating 10 experimental conditions. The experiment evaluated factors at two levels of temperature (30 and 60 °C), pressure (15 and 45 MPa), and ethanol percentage as a cosolvent (0 and 30% *v*/*v*). The conditions at the central point were 45 °C, 30 MPa, and 15% *v*/*v* ethanol. The impact of these factors on response variables such as extraction yield (Yield), total phenolic content (TPC), and antioxidant activity (TEAC) was determined in triplicate using tola biomass (n = 3). The analyses of these compounds and antioxidant activity will be described in the following sections.

Multi-response optimisation was performed using the desirability function approach to determine the optimal extraction conditions. The optimisation criteria were set to maximise yield, TPC, and TEAC simultaneously. Individual desirability functions (di) were calculated for each response and combined into a global desirability index (D) using a geometric mean. The resulting mathematical models were used to generate response surface plots, and the predicted optimal conditions were subsequently validated through independent experimental replicates to ensure the model’s reliability.

### 2.7. Mathematical Modelling: Linear Spline Model

A crucial step in designing efficient SFE or pressurised liquid (PLE) methods is to model the kinetic behaviour of the extraction process. Typically, a variable is chosen to track the cumulative mass of the extract versus extraction time or solvent consumption. This representation, known as the Overall Extraction Curve (OEC), is widely used and obtained by plotting extraction time versus the accumulated extract. An example of a simplified approach to modelling the extraction curve is the linear spline model, as presented by Meireles [22]. The extraction was performed under the conditions specified by the centre–point of the experimental design (45 °C, 30 MPa, and 15% *v*/*v* ethanol). Extracts were collected every 5 min for the first 30 min, then every 10 min until 70 min, and then every 20 min until 150 min; the final extract was collected at 180 min. Each curve comprised 15 extraction points. The extraction yield (Y, % *w*/*w*) was determined at each point on the curve. This experiment was performed in duplicate. Notably, a kinetic study was not conducted under SFE-CO2 conditions without the ethanol cosolvent, as none of these runs is expected to yield results exceeding those obtained with the cosolvent.

The OEC was modelled using a linear spline approach (Equation (1)) and segmented into three linear sections as defined by Equations (2)–(4). The fitting process was carried out using PROC REG and PROC NLIN in SAS 9.04 (SAS University Edition, SAS Institute Inc., Cary, NC, USA). Each segment corresponded to a distinct extraction phase governed by different mass transfer mechanisms: the constant extraction rate (CER) phase and the FER phase, the latter encompassing both convection-driven and diffusion-controlled (DC) regimes, following the framework proposed by Meireles [22]. From the spline model, the mass transfer rate during the CER phase (MCER) and the transition time between CER and FER phases (tCER) were extracted. An analogous method was employed to analyse the FER phase, thereby delineating the DC phase. The experimental OEC data were thus successfully fitted, and the equilibrium mass ratio of the solute in the supercritical phase at the outlet (YCER) was estimated by dividing the MCER value by the average solvent flow rate recorded during the CER stage. Similar calculations were performed for the FER and DC periods to obtain Y_FER_ and Y_DC_ and M_FER_ and M_DC_.

(1) $$y=m_{Ext}=\left(b_{0}-\sum_{i=1}^{i=N}C_{i}a_{i+1}\right)+\sum_{i=1}^{i=N}a_{i}t$$

For one straight line:

(2) $$y=m_{Ext}=b_{0}+a_{1}t\;for\;t\;\leq \;t_{CER}$$

For two straight lines:

(3) $$y=m_{Ext}=b_{0}-t_{CER}a_{2}+\left(a_{1}+a_{2}\right)\;t\;for\;t_{CER}<t\;\leq \;t_{FER}$$

For three straight lines:

(4) $$y=m_{Ext}=b_{0}-t_{CER}a_{2}-t_{FER}a_{3}+\left(a_{1}+a_{2}+a_{3}\right)\;t\;for\;t_{FER}<t$$

where y = response variable; $m_{Ext}$ indicates the mass of the extract; b_o_ = linear coefficient; $a_{i}$ (i = 1, 2, 3); are the intercepts of these lines (for example, $a_{1}\;$ is the intercept of the first and second lines, and $a_{2}$ is the intercept of the second and third lines). t = time (min); $t_{CER}$ = CER time (min); $t_{FER}$ = FER time (min).

Using the adjusted parameters, the Y_CER_, Y_FER_, and Y_DC_ achieved in t_CER_, t_FER_, and t_DC_ were calculated. Then, the recovery (%) was calculated at each time point using Equation (5).

(5) $$Recovery\;\left(\%\right)=\frac{y_{t}}{y_{\;Total\;time}}\times 100$$

### 2.8. Total Phenol Content (TPC)

TPC was determined using the 96-well microplate Folin–Ciocalteu method previously described in detail by Ruiz-Domínguez et al. [23]. A microplate reader (BioTek Synergy HTX multi-mode reader; Gen 5 2.0 software, Winooski, VT, USA) was used to measure the corrected absorbance at 765 nm. To calibrate, standards of different concentrations of gallic acid (ranging from 0 to 2 mg/mL) were utilised. The results were expressed as mg of gallic acid equivalents (GAE)/g of extract and were presented as an average of three measurements.

### 2.9. Confocal Laser Scanning Microscopy

Exploiting the intrinsic fluorescence properties of phenolic compounds [24], tola plant biomass samples were characterised pre- and post-optimal SFE treatment for TPC (performed at 60 °C, 15 MPa, and 30% ethanol, corresponding to run 7). Imaging was conducted using a TCS SP8 inverted confocal laser scanning microscope (Leica Microsystems, Wetzlar, Germany) equipped with a 40× objective lens. Excitation was induced with a 488 nm laser line (blue spectrum), and fluorescence emission was collected in the 499–551 nm range (green channel), enabling visualisation and comparative analysis of phenolic compound distribution.

### 2.10. Determination of Antioxidant Activity

The TEAC value was determined using the method described by Re et al. [25], with a few modifications. First, 2,2′-azino-bis (3-ethylbenzothiazoline-6-sulfonic acid) diammonium salt (ABTS^•+^) radical was produced by reacting 7 mM ABTS and 2.45 mM potassium persulfate in the dark at room temperature for 16 h. The resulting aqueous ABTS^•+^ solution was then diluted with 5 mM sodium phosphate buffer (pH 7.4) to an absorbance of 0.70 ± 0.02 at 734 nm. Subsequently, 20 µL of the sample was mixed with 180 µL of the ABTS•^+^ solution in a 96-well microplate, and the absorbance of the reaction was measured at 734 nm over 10 min using a spectrophotometer. Trolox (6-hydroxy-2,5,7,8-tetramethylchromane-2-carboxylic acid) was used as the reference standard. Results were expressed as TEAC values, in mmol Trolox equivalents (TE) per g of extract. All measurements were performed in triplicate (n = 3).

### 2.11. Extraction of Fatty Acids

FAMEs were extracted using a direct acid-catalysed transesterification method. Briefly, 10 mg of SFE extract (or biomass) were combined with 10 ppm of an internal standard and 3 mL of 5% *v*/*v* H_2_SO_4_ in methanol. The mixture was incubated at 80 °C for 1 h under constant agitation. After incubation, the reaction mixture was washed with hexane and Milli-Q water until the aqueous phase reached neutral pH. Phase separation was performed by centrifugation at 360× *g* for 10 min at room temperature. The upper hexane layer, containing the FAMEs, was collected, analysed and quantified by GC–MS.

### 2.12. Analysis and Quantification by Gas Chromatography–Mass Spectrometry (GC–MS)

Transesterified samples from MTB were analysed by injecting 1 µL into a Q Exactive™ GC Orbitrap™ mass spectrometer (Thermo Fisher Scientific, Waltham, MA, USA) equipped with a Zebron-FAME column (60 m × 0.25 mm i.d., 0.20 µm film thickness; Phenomenex, Torrance, CA, USA). The oven temperature program was as follows: initial temperature at 100 °C (held for 3 min), ramped at 2.5 °C/min to 240 °C, and held at 240 °C for 10 min. The injector temperature was set to 250 °C. Helium was used as the carrier gas at a constant flow rate of 1.5 mL/min in splitless mode. Compound identification was performed by comparing the acquired mass spectra with entries from the NIST Mass Spectrometry Data Center (MS 2.3 software version) and by matching retention times with those of a commercial FAME standard mix (FAME Mix C4–C24, Supelco Analytical, Bellefonte, PA, USA). The relative quantification of FAMEs was performed by summing the peak areas of all detected FAMEs in each chromatogram. Individual fatty acids were expressed as their percentage contribution to the total FAME peak area (*p* < 0.05; n = 3; SD ≤ 5%).

### 2.13. Statistical Analysis

All experiments were designed, and data were analysed using a screening design approach. The specific design used was a Screening design, specifically Factorial 2^3^ with Resolution V^+^. This design included two centre points and four response variables, and the analysis was performed using the Statgraphics Centurion XVIII^®^ (StatPoint Technologies, Inc., Warrenton, VA, USA) software (Version XVIII). The order of the experiments was fully randomised to mitigate the effects of lurking variables. The effects of the factors on the response variables in the separation process were assessed using pure error, considering a 95% confidence interval for all variables. Statistical significance was determined using analysis of variance (ANOVA) and the standardised Pareto chart. Additionally, response surfaces for the respective mathematical models were obtained, and a *p*-value ≤ 0.05 was considered significant. In this specific design involving three factors (X_1_, X_2_ and X_3_), the relationship between the response variable and these factors can be approximated by a second-degree quadratic polynomial model (Equation (6)). The general mathematical representation is expressed as follows:

(6) $$Z=\beta_{0}+\beta_{1}X_{1}+\beta_{2}X_{2}+\beta_{3}X_{3}+\beta_{12}X_{1}X_{2}+\beta_{13}X_{1}X_{3}+\beta_{23}X_{2}X_{3}+\beta_{11}X_{1}^{2}+\beta_{22}X_{2}^{2}+\beta_{33}X_{3}^{2}$$

where Z represents the estimated response, β_0_ is the constant term, β_1_, β_2_ and β_3_ are the linear coefficients, β_12_, β_13_, and β_23_ denotes the interaction coefficient between factors X_1_, X_2_, and X_3_, and β_11_, β_22_, and β_33_ correspond to the quadratic coefficients. All measurements were performed in triplicate (n = 3). Furthermore, a multi-response analysis using the desirability function approach was applied to evaluate the response variables jointly. For the remaining results, significant differences were evaluated using Duncan’s multiple–range test (MRT) at the 95% confidence level (*p* ≤ 0.05).

## 3. Results and Discussion

### 3.1. Identification and Phylogenetic Analyses

The genus was identified by amplifying and sequencing the nuclear ribosomal internal transcribed spacer (ITS) region [12,14] with two independent primer pairs targeting partially overlapping fragments, as described in Section 2. Given the morphological similarity among *Parastrephia* species, the use of two primer sets ensured sequence consistency and strengthened taxonomic reliability. BLAST (https://blast.ncbi.nlm.nih.gov/) analyses against the National Center for Biotechnology Information (NCBI) database indicated 100% query coverage and 100% sequence identity with sequences deposited as *Parastrephia quadrangularis* and *Parastrephia lepidophylla*, both species native to the Chilean Andean Altiplano [3], where samples were collected. These results confirm the placement of the specimens within the genus *Parastrephia*.

**Figure 2:**
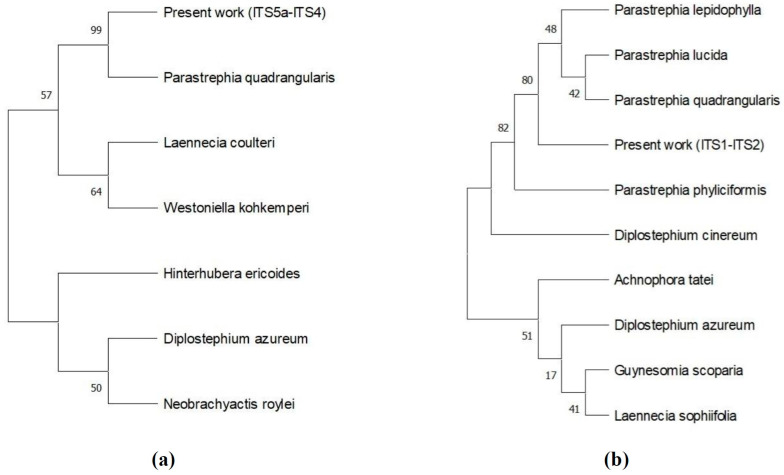
Maximum likelihood phylogenetic trees based on nuclear ribosomal ITS sequences: (**a**) Phylogenetic reconstruction inferred from the ITS5a–ITS4 fragment. (**b**) Phylogenetic reconstruction inferred from the ITS1–ITS2 fragment. Bootstrap values (>50%) are shown at the nodes.

Maximum likelihood phylogenetic analyses based on the two ITS datasets further supported these findings (Figure 2). The tree inferred from the ITS5a–ITS4 fragment (Figure 2a) showed a strongly supported sister relationship between the sequences obtained in this study and *P. quadrangularis* (bootstrap = 99), providing clear species-level resolution. The phylogeny reconstructed from the ITS1–ITS2 fragment (Figure 2b) also placed the sequences within the *Parastrephia* clade; however, it exhibited lower internal resolution among closely related species. Importantly, both ITS datasets yielded congruent topologies and consistently supported the placement of the specimens within *Parastrephia*. Taken together, the concordant results from two partially overlapping ITS fragments, combined with strong phylogenetic support in the ITS5a–ITS4 analysis and geographic consistency with the species’ known distribution, support the identification of the specimens as *P. quadrangularis*.

### 3.2. Conventional Extraction

Two green solvents, including water and ethanol, were used to find the most effective extractant for obtaining high yield, total phenol content (TPC, measured in mg GAE/g extract), and antioxidant activity expressed as Trolox equivalent (TE) antioxidant capacity or TEAC in mmol TE/g extract from tola herb (Table 1). Yield varied across all cases with significant differences (*p* < 0.05), and the highest extract yield (31.73 ± 0.26) was recovered using only ethanol. Because yield is directly related to the efficiency of the extraction process, it is mainly influenced by the nature of the solvent used [11]. In a study conducted on the genus *Parastrephia*, Cifuentes et al. [26] extracted for 72 h at room temperature using ethanol:water (1:1) as the solvent. The obtained extraction yield was 26% (*w*/*w*), which was 5% lower than the yield obtained in our study for 100% ethanol; however, it should be considered that various environmental, growth, and cultivation conditions of plants can affect the overall synthesis of compounds in a given species [27]. Plaza-Cazón et al. [6] employed water as a solvent, following a natural leaching extraction approach, rather than relying on organic solvents or extraction methods unsuitable for field applications. Even though they obtained lower yields than ours (about 13%, *w*/*w*), their extracts were rich in bioactive compounds with antimicrobial activity, including euparin (benzofurans) and phenolic acid (ferulic acid). It has been shown that pre-treating a sample before analysis can increase or decrease the number of compounds released. Metrouh Amir et al. [28] also compared the use of different solvents (water, acetone, methanol, ethanol, and their 50% aqueous dilutions) in extraction (3 h at room temperature) for *Matricaria pubescens* (Asteraceae) biomass. The highest yield, 34.68% (*w*/*w*), was obtained using an ethanol:water (1:1) mixture. Accordingly, they found many polar compounds in the extracts, as we predicted in this study.

The TPC showed significant differences among the two types of solvents used (*p* < 0.05, Table 1). The highest concentration was obtained when 100% ethanol was used as the extractant (21.74 ± 0.20 mg GAE/g extract). Our findings aligned with those of Casagrande et al. [8], who quantified the TPC of the extract obtained by maceration of *Braccharis dracunculifolia* (Asteraceae) at temperatures ranging from 40 to 80 °C. They found the highest TPC when using 100% ethanol and acetone (21.20–33.87 and 22.09–37.22 mg GAE/g extract, respectively), regardless of the extraction temperature. Ardiles et al. [29] extracted bioactive compounds from dried, chopped aerial parts of *P. quadrangularis* collected in northern Chile. The extraction was performed using absolute ethanol (24 h, repeated three times in darkness), yielding 36 g of extract, corresponding to approximately 7.2% (*w*/*w*). Ultra-high-performance liquid chromatography coupled with quadrupole-Orbitrap mass spectrometry (UHPLC-Q-Orbitrap) was employed to identify metabolites in their extracts, including phenolic acids and flavonoids (e.g., euphorbetin, caffeic acid, kaempferol, isorhamnetin and eriodictyol), which are associated with gastroprotective activity. On the other hand, Metrouh Amir et al. [28] demonstrated that the concentration of phenolic compounds in *Matricaria pubescens* (Asteraceae) varies depending on the solvent used. The highest phenolic compound concentrations, at 2.65 mg GAE/g extract, were observed in extracts prepared with 50% methanol and 50% ethanol. These results allowed us to conclude that plants in the Asteraceae family are rich in phenolic compounds. Considering that the recovery of the phenolic compounds of our MTB was higher when a higher concentration of ethanol was used for extraction (30%), we concluded that this solvent could more efficiently dissociate the phenolic compounds from the interfering substances. Thus, considering the relevant bioactive nature of plant phenolic compounds, namely anti-inflammatory activity, and their chemical diversity and polarity, mixtures of both miscible polar and moderately non-polar solvents, e.g., water or methanol plus ethanol, could address a more efficient extraction of phenolic compounds to recover as much bioactivity as possible.

The antioxidant activity described in Table 1, calculated as TEAC, was highest when water was used as the solvent (2.54 ± 0.23 mmol TE/g extract), followed by the extract obtained with ethanol (2.39 ± 0.09 mmol TE/g extract). There was no significant difference between them. The type and composition of the solvent had a significant impact on the antioxidant activity, as the OH-radicals responsible for the activity were influenced by the solvent, leading to concentration variations in the analyte [30]. Based on these findings, the use of 100% ethanol yielded a higher concentration of polyphenols (21.74 ± 0.20) compared to using 100% water (8.05 ± 0.08) (which reduced the antioxidant activity). A similar pattern of variation was also reported by Metrouh Amir et al. [28] for *M. pubescens* (Asteraceae). In that study, significant differences (*p* < 0.05) were recorded in the antioxidant activity for all solvents, with the highest activity detected in the extract obtained with ethanol (32.30 ± 0.46 IC_50_ mg/mL), followed by the extract obtained with water (17.78 ± 0.32 IC_50_ mg/mL), Casagrande et al. [8] also found a variation in the antioxidant activity based on the solvent used to extract *B. dracunculifolia* (Asteraceae) samples. The highest antioxidant activity was obtained with acetone (202.21–399.67 µmol TEAC/g extract), followed by the ethanolic extract (167.03–320.00 µmol TEAC/g extract) and the aqueous extract (140.26–267.70 µmol TEAC/g extract). The total phenolic content (TPC) of an extract does not always exhibit a direct proportional relationship with its antioxidant activity. This variability arises because antioxidant activity is determined not only by phenolic concentration but also by the chemical structure of phenolic compounds [31]. The extraction efficiency of specific antioxidant groups is influenced by the solvent used, which selectively favours certain compounds [30]. These differences ultimately impact the quantification of antioxidant activity [32]. The selective extraction of highly bioactive compounds, or extracts enriched in them, should drive efforts to develop experimental strategies that yield high-bioactivity extracts, a step toward formulating commercial products.

### 3.3. Supercritical Fluid Extraction (SFE)

SFE experiments were conducted using ethanol, identified as the most effective polar solvent in the conventional extraction section (Table 2), in combination with CO_2_, an apolar solvent. This approach aimed to identify bioactive molecules present in tola extracts. For this purpose, a factorial design (2^3^) with two centre points was employed to optimise the extraction process, assessing variables such as extraction yield, total phenolic content (TPC), Trolox equivalent antioxidant capacity (TEAC), and the fatty acid (FA) profile of *P. quadrangularis* biomass. Additionally, a linear spline model was used to determine the optimal extraction time based on the extraction yield variable.

#### 3.3.1. Mathematical Modelling and Kinetic Curve: Linear Spline

The specific conditions chosen for the kinetic study were based on the values located at the centre point of the experimental design. These conditions were: 45 °C, 30 MPa, and CO_2_:ethanol flow rates 85:15 *v*/*v* ratio. These conditions were selected to avoid extreme levels of each selected factor so that the work surface was equidistant from the borders. This approach enabled the SFE to obtain performance results from the MTB. Moreover, ethanol, a cosolvent, was proposed for SFE as the best extractant based on prior conventional extraction experiences.

**Figure 3:**
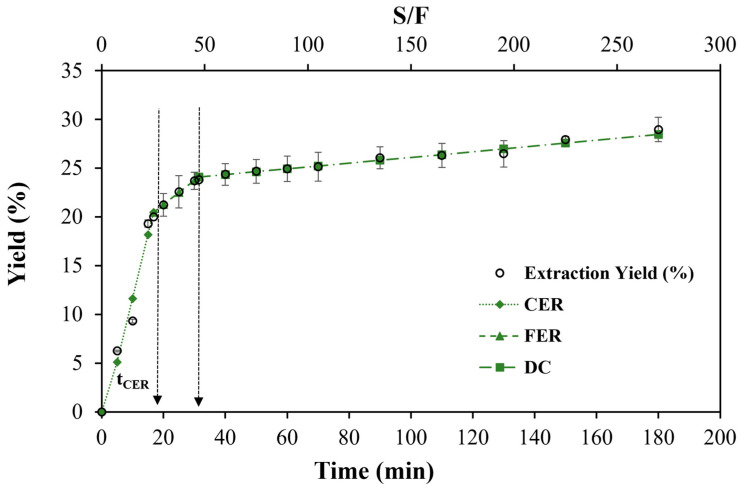
Overall extraction curves (OEC) of the tola biomass by SFE at P = 30 MPa, T = 45 °C, and CO_2_ + ethanol (85:15 *v*/*v*) flow rate of 3.31 g/min.

The OECs obtained from the dry biomass of *P. quadrangularis* are shown in Figure 3. A total cumulative extract yield of 28.97% was obtained at the end of 180 min of extraction, for which ~2.0 g of dry biomass was used. The OEC graph for dry biomass exhibited SFE kinetic behaviour similar to that reported by Wrona et al. [33], Ruiz-Domínguez et al. [23], and Salinas et al. [21], who utilised plant material, microalgae, and fruit (*chañar*), respectively, as biomass resources. The extraction process began with the CER period, characterised by rapid extraction of easily recoverable bioactive compounds using CO_2_ as solvent and ethanol as the cosolvent. The CER period is primarily influenced by the unit operation of convection-type mass transfer in the fluid layer surrounding the biomass particles. At the end of the CER period, a second transition period began: the FER period. During this period, the extraction rate slows and is controlled by mechanisms typical of mass transfer. In the third and final stage, the solute extraction rate was considerably slower because access of the extracting solvents to the interior of the herbaceous biomass matrix became limited. In the final stage, the diffusion between biomass particles is the main mechanism of SFE mass transfer. This stage is commonly called DC (diffusion-controlled), and its typical behaviour is a slight diffusion curvature with a minimum extraction rate [34].

The OEC parameters (Table 3) were calculated from the data obtained and adjusted using the spline linear model (Figure 3). The t_CER_ was 16.78 min with accumulated extracts of 20.01% and a recovery rate of 69.05%. At this stage, the CO_2_ + ethanol (85:15 *v*/*v*) flow rate reached a solvent-to-feed ratio (S/F) of 27.51, with a superficial velocity of 0.19 mm/s. This result was consistent with findings from other studies, which reported recovery rates of 50–90% during the CER period [35,36]. The t_FER_ was 31.42 min, with S/F = 51.53; after excluding the CER period, the t_FER_ was only 14.64 min. Thus, the accumulated extract in FER was only 3.79%, and a recovery of 13.10% was recorded. Short FER periods occur in herbaceous materials such as *Nigella indica*, whose FER period at 50 MPa and 40 °C was found to be 12–17 min [33], as well as in plants such as marigolds (*Calendula officinalis*), whose FER period at 34 MPa, 40 °C, and flow rates of 30 g/min (10 times higher than those values shown in this study) was only 20 min [37]. In our study, a total recovery (CER + FER) of 23.80% was recorded, with a total recovered extract of 82.15% (Table 3).

The M_CER_ and M_FER_ values represent the extraction rates during the CER and FER periods, respectively, at 2.4 × 10^−2^ and 5.45 × 10^−3^ g/min (Table 3). These values were lower than those reported for the M_CER_ and M_FER_ of *chañar* almond oil extracts of 2.4 × 10^−2^ and 1.24 × 10^−2^ g/min, respectively [21]. When expressed in Y = mg extract/g biomass, the results were 199.94 and 49.42 for the CER and FER periods, respectively, which represented in Y* = g extract/g _(CO2 85% + ethanol 15%)_, 7.3 × 10^−3^ and 1.7 × 10^−3^ for both periods. However, in the final DC stage, the M_DC_ and Y*_DC_ were lower than their corresponding values in the two previous periods. Nevertheless, the Y_DC_ exceeded the Y_FER_ but was lower than the Y_CER_. This was due to the extent of the last stage during the FER period, a recurring behaviour of the OEC in herbaceous plants [33]. In summary, OEC behaviour across the three periods was observed, with the highest extraction rate occurring in the CER period; these findings were supported by previous studies [22,38]. Additionally, the OEC exhibited behaviour expected for SFE kinetics in plant matrices, as reported by Venturi et al. [39].

#### 3.3.2. Optimisation of Extraction Yield

The results described in this section are presented in Table 1. For the extraction yield, the optimal extraction was calculated using the linear spline model (40 min), corresponding to a time greater than 8.58 min above the tFER, with a recovery of 84.01%. The supercritical behaviour of each response variable is described below. In particular, the three highest extraction yields were 34.95%, 33.71%, and 33.40% *w*/*w* (runs 9, 8, and 6, respectively) (Table 1). These results were closely aligned with the optimal conditions predicted by the Statgraphics Centurion software (version XVIII), which estimated a maximum extraction yield of 35.88% (*w*/*w*) under 60 °C, 45 MPa, and 30% *v*/*v* ethanol as the cosolvent. In all three experimental cases, the highest cosolvent concentration (30% *v*/*v* ethanol) was utilised. The design matrix, analysed using statistical software, generated the general equation (Equation (7)) for the working model, as follows:

(7) $$\begin{array}{l} Y=10.1965-0.0923333\times T-0.1965\times P+1.01667\times Ethanol\\ +0.00563333\times T\times P-0.00434444\times T\times Ethanol\\ +0.00161111\times P\times Ethanol \end{array}$$

**Figure 4:**
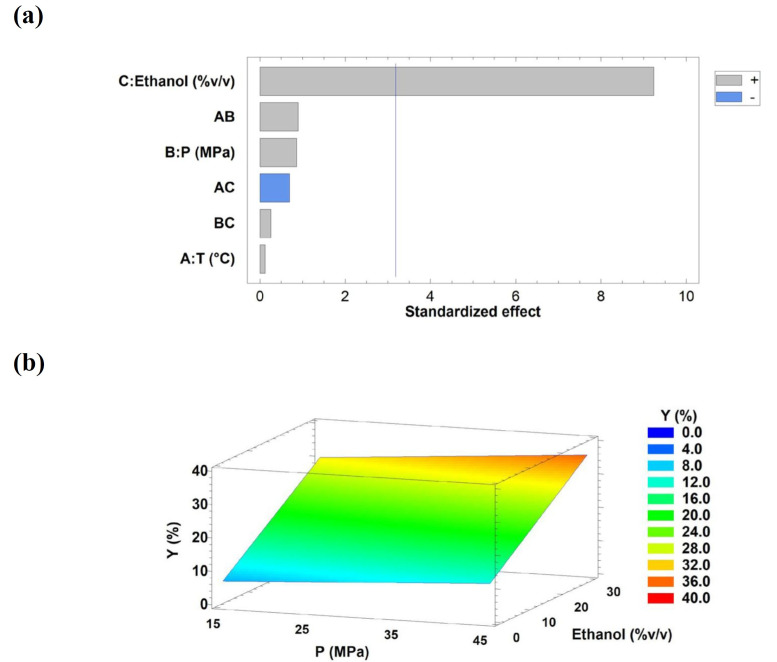
Pareto chart (**a**) and Response Surface Curve (**b**) of the combined effects of temperature (30–60 °C), pressure (15–45 MPa), and ethanol as a cosolvent (0–30% *v*/*v*) on the extraction yield from the tola biomass. Response surface curves were drawn at the optimal temperature (60 °C). Abbreviation: temperature (T) and pressure (P).

In this model, Y represents the extraction yield (%, *w*/*w*), T denotes temperature (°C), P represents pressure (MPa), and Ethanol corresponds to the percentage of cosolvent with CO_2_. The standard deviation of the extraction yield was 96.83%. Based on the regression coefficients and *p*-values from the 2^3^ factorial design (Table S1, Supplementary Materials) and the Pareto chart (Figure 4a), only ethanol as a cosolvent was statistically significant in the model. Consequently, the non-significant terms were removed from the general equation (Equation (7)), yielding the simplified expression Equation (8), with an R^2^ value of 94.35%.

(8) Y=7.7515+0.8695×etanol

The Pareto chart and response surface plots were generated to evaluate the effects and interactions of the independent variables on the extraction yield (Y) and to determine the optimal operating conditions (Figure 4). Ethanol was the determining factor in obtaining the highest extraction yields (*p* < 0.05). Increasing the cosolvent in the extraction cell increased the yield. Using response surface methodology (RSM; Figure 4b), a maximum extraction yield close to 36% *w*/*w* was predicted at high temperature (60 °C) and pressure, with 30% *v*/*v* ethanol as the cosolvent. Other factors, such as temperature, pressure, and their combinations, did not impact the optimisation process. However, yields obtained under CO_2_ as the solvent were the lowest in this study (ranging from 5.26% to 9.78%, *w*/*w*, runs 2–5). Similar findings were reported in other studies on matrices known to contain chemical compounds with anti-inflammatory and antioxidant properties (with some degree of polarity) [40]. The results showed that the temperature and pressure did not significantly influence the yield obtained from the MTB.

We compared the extraction yields obtained from tola by using SFE (30% ethanol) versus conventional extraction using 100% ethanol, and no significant differences were found. In contrast, the use of water:EtOH mixture resulted in a considerable difference in the SFE results. This can be explained by differences in the solvents’ polarity, which can enhance the recovery of target compounds [41]. Based on the linear spline model, our SFE experiments were performed for 40 min, compared with the 24 h solvent extraction process. Messina et al. [42] showed that a product free from toxic solvents can be obtained via supercritical extraction. Additionally, this process has a lower environmental impact and is inexpensive and quick. These are qualities that are strongly demanded by the industry to minimise production costs.

#### 3.3.3. Total Phenolics Content (TPC)

The TPC obtained in the supercritical extracts is presented in Table 1. We highlighted the highest concentrations obtained with 30% *v*/*v* ethanol as the cosolvent. The highest TPC values were 27.90 and 25.08 mg GAE/g extract (runs 7 and 9, respectively), obtained at 15 and 45 MPa, respectively, at an extraction temperature of 60 °C. These results indicated that increased TPC concentrations in tola extracts can be obtained by using the highest temperature setting and the highest cosolvent percentage. The optimal conditions determined by the statistical software closely matched the experimental results, yielding a TPC of 8.95 mg GAE/g extract at 60 °C, 15 MPa, and 30% *v*/*v* ethanol as a cosolvent. Based on the 2^3^ factorial design with central points, the TPC was accurately represented by the model described in Equation (9), with an R^2^ coefficient of 95.11%.

(9) $$\begin{array}{l} TPC=10.603-0.211667\times T-0.388667\times P+0.551\times Ethanol+\\ 0.0132444\times T\times P+0.00902222\times T\times Ethanol-0.0173556\times P\times Ethanol \end{array}$$

**Figure 5:**
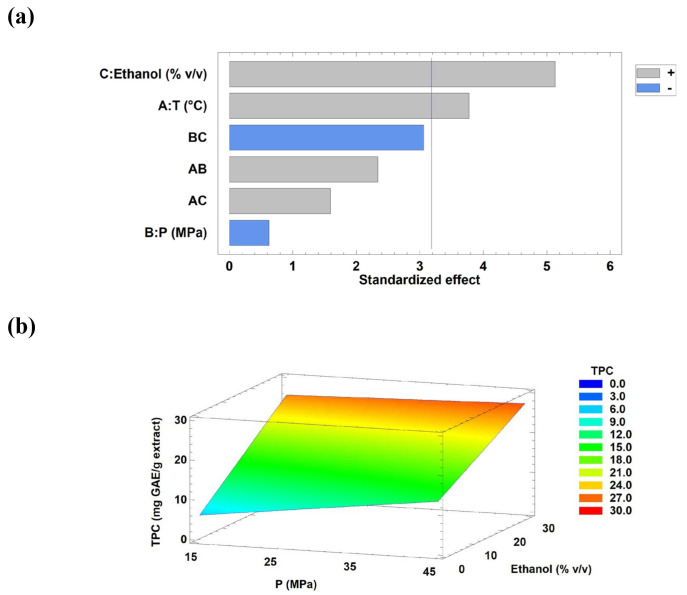
Pareto chart (**a**) and Response Surface Curve (**b**) of the combined effects of temperature (30–60 °C), pressure (15–45 MPa), and ethanol as a cosolvent (0–30% *v*/*v*) on TPC from the tola biomass. Response surface curves were drawn at the optimal temperature (60 °C). Abbreviation: temperature (T) and pressure (P).

In this model, TPC signifies the total phenolic content (mg GAE/g extract), T represents temperature (°C), P indicates pressure (MPa), and Ethanol refers to the percentage of cosolvent with CO_2_. Based on the regression coefficients and *p*-values obtained from the factorial design (Table S1, Supplementary Materials) and the Pareto chart (Figure 5a), ethanol as a cosolvent and temperature were identified as statistically significant factors in the model. As a result, the non-significant terms were excluded from the general equation, leading to the simplified expression (Equation (10)), which exhibited a higher deviation (R^2^ = 66.16%).

(10) TPC=−7.147+0.321×T+0.436333×Ethanol

The above findings were consistent with the Pareto and response surface plots (Figure 5). The percentage of ethanol as the cosolvent was a significant factor in recovering a higher TPC from tola. As phenolic compounds are generally polar (with exceptions), increasing the fraction of ethanol or another more polar cosolvent, coupled with CO_2_-only extraction, can increase the final recovered concentration of phenols. This is consistent with our results (Table 1), where most of the lower concentrations of phenolic compounds were obtained in extractions without the coupled cosolvent (CO_2_ solvent).

Although the individual effects of temperature and pressure on the extraction process (Y) were not statistically significant, a slight improvement in phenolic content was observed at higher levels of both factors, regardless of the presence of the cosolvent. At the lowest pressure evaluated (15 MPa), the response surface (Figure 5b) shows that increasing temperature and ethanol concentration markedly enhance TPC, suggesting that solvent polarity and thermal effects play a dominant role in phenolic extraction under low-pressure conditions. Casagrande et al. [8] and Tušek et al. [9] performed the extraction of *B. dracunculifolia* and four herbs belonging to the Asteraceae family (i.e., chamomile, dandelion, calendula, and yarrow) by maceration. They reported significant differences in the TPC at temperatures from 40 to 80 °C. These results indicated that the Asteraceae family is rich in phenolic compounds, which can be more efficiently extracted at high temperatures. This might be associated with an increase in the solubility of target compounds, thereby favouring the separation of phenolic compounds from interferents such as carbohydrates and proteins, among other compounds present in the samples [43]. The pressure exerted is another important factor reported by several studies on SFE. These studies have shown that higher pressure can increase the final concentration of phenolic compounds in extracts of different natural raw materials [44,45]. Increasing the pressure can directly increase solvent density and enhance the solvent’s extraction effectiveness.

**Figure 6:**
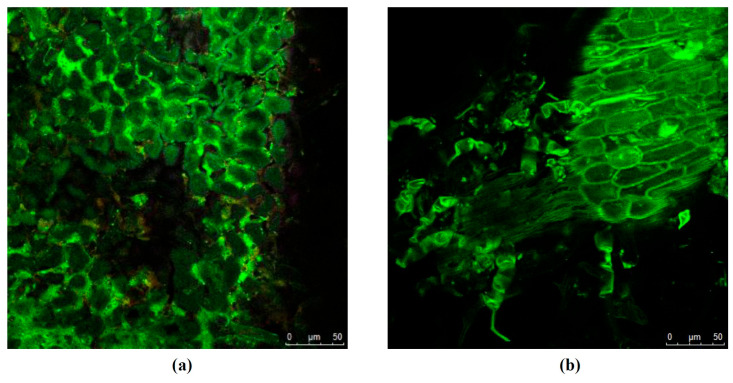
Confocal micrographs of the tola biomass (**a**) before extraction; (**b**) after the best condition of SFE for TPC (60 °C/15 MPa/30% ethanol).

To confirm the presence of phenolic compounds in tola and assess extraction efficiency, confocal micrographs of the dried plant biomass were acquired before and after SFE (Figure 6), based on the intrinsic autofluorescence of polyphenolic compounds [24]. Confocal autofluorescence microscopy was used solely as a qualitative tool to corroborate the presence of phenolic compounds and was not intended for fluorescence-based quantification.

The SFE condition selected for this evaluation was the best experimental condition for TPC recovery (Table 1, run 7; TPC equal to 27.90 mg GAE/g extract). The biomass without pretreatment of supercritical extraction is shown in Figure 6a. As shown in the image, the presence of TPC in tola was very intense. Additionally, the cells remained intact even after freeze-drying. In contrast, slightly lower fluorescence emission in the green region (500–530 nm) confirmed the presence of polyphenols, flavonoids, and/or tannins [24] in tola cells after supercritical extraction, thereby providing evidence of partial extraction (Figure 6b).

The confocal microscopy study evidenced that the extraction method, conditions, and solvent choice were improved. Other researchers have suggested using green technologies, such as PLE or ultrasound-assisted extraction, as suitable methods for solubilising polar compounds, including phenolic molecules [46]. To improve the extraction efficiency of target compounds from different natural sources, some researchers have proposed using multiple extraction methods sequentially [47].

Deformation and rupture of the tola cell wall caused by the high working pressures of the supercritical technique (Figure 6b). Indeed, this technology can promote cell wall rupture, increasing solvent penetration into the extracted biomass and thereby increasing target compound extraction yields and, in some cases, selectivity. These extraction conditions should be improved in future studies to maximise the TPC recovery.

#### 3.3.4. Antioxidant Activity

**Figure 7:**
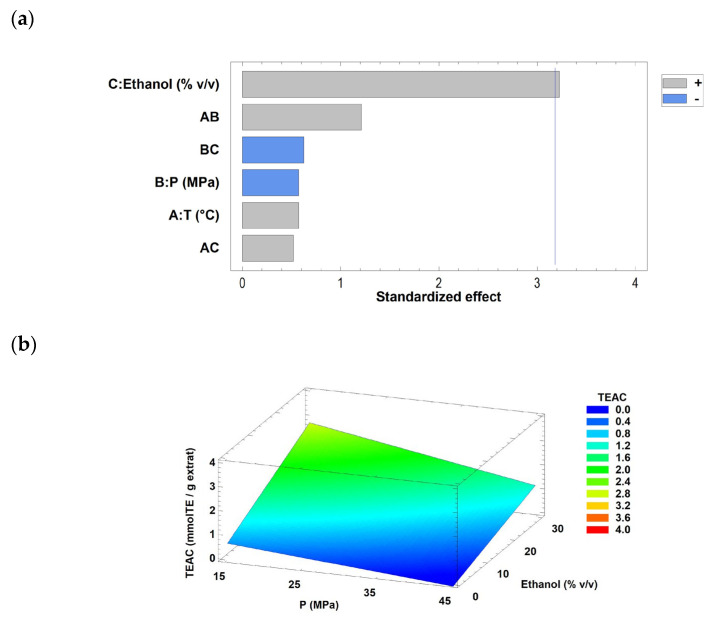
Pareto chart (**a**) and Response Surface Curve (**b**) of the combined effects of temperature (30–60 °C), pressure (15–45 MPa), and ethanol as co-solvent (0–30% *v*/*v*) on TEAC from the tola herb. Response surface curves were drawn at the optimal temperature (30 °C). Abbreviations: temperature (T) and pressure (P).

The antioxidant activity of tola supercritical extracts, measured by TEAC, is also presented in Table 1. The highest values of antioxidant activity obtained were 2.98 and 2.91 mmol TE/g extract (runs 6 and 9, respectively), obtained under different conditions of temperature, pressure, and cosolvent percentage (i.e., 30 °C, 15 MPa, and 30% ethanol and 60 °C, 45 MPa, and 30% ethanol, respectively). The optimal conditions for maximising TEAC as the response variable in the factorial design for MTB extraction were determined using statistical software. These conditions were at 30 °C, 15 MPa, and 30% *v*/*v* ethanol as cosolvent. The maximum TEAC value obtained was 2.78 mmol TE/g extract, closely aligning with the experimental results. The regression model fitted to the data (Equation (11)) has an R^2^ of 81.47%.

(11) $$\begin{array}{l} TEAC=2.52525-0.052\times T-0.0785833\times P+0.0641667\times Ethanol+\\ 0.00177222\times T\times P+0.000761111\times T\times Ethanol-0.000916667\times P\times Ethanol \end{array}$$

where TEAC represents the antioxidant activity (mmol TE/g extract), T is the temperature (°C), P is the pressure (MPa), and Ethanol corresponds to the percentage of cosolvent in CO_2_ (% *v*/*v*). The Pareto chart (Figure 7a) indicated that ethanol as a cosolvent was the only statistically significant factor (*p* < 0.05) affecting antioxidant activity. This effect is illustrated in the response surface plot (Figure 7b), which shows the influence of ethanol content on TEAC across the experimental domain. Accordingly, increasing the solvent ratio was associated with enhanced antioxidant activity in tola extracts obtained by SFE.

To simplify interpretation, the mathematical model was subsequently refined by retaining only the significant variable (ethanol as a cosolvent) and removing non-significant terms, yielding the adjusted equation (Equation (12)) with an R^2^ of 64.30%.

(12) TEAC=0.22025+0.0709167×Ethanol

Antioxidant activity increases with increasing extraction temperature and phenolic compound concentration. Although pressure did not strongly affect the antioxidant activity, it was still considered because any change can modify the detected antioxidant activity [44]. After analysing these results, we concluded that maintaining the highest cosolvent concentration would yield an extract with high antioxidant activity, even when varying pressure and/or temperature. This is because most of the antioxidant activity is exerted by the presence of polar compounds in the *P. quadrangularis* biomass.

Several studies reported these trends. Messina et al. [42] compared different extraction procedures of bioactive compounds from four *Calendula* spp. They performed extractions by maceration and by SFE using only CO_2_ as the solvent and 95% ethanol with a coupled cosolvent. Their results showed that the highest antioxidant activity was achieved with SFE of *Calendula maritima* using 95% ethanol. The researchers concluded that although better results were obtained with solvent-maceration extractions, the results obtained using the SFE method were similar. Villalva et al. [48] performed ultrasound-assisted extractions using 50% and 100% ethanol as solvents, as well as CO_2_ supercritical extraction. They found that using more polar solvents led to the extracts of *P. quadrangularis* with higher antioxidant activity, mainly due to the presence of total phenols.

#### 3.3.5. Multiple Response Optimisation

As previously described by Derringer et al. [49], multi-response optimisation based on desirability functions is a robust methodology for simultaneously optimising multiple responses. In this context, its application to *P. quadrangularis* identified extraction conditions that simultaneously maximised extraction yield, TPC, and antioxidant capacity, highlighting the suitability of this methodology for valorising underexplored native plant resources.

**Figure 8:**
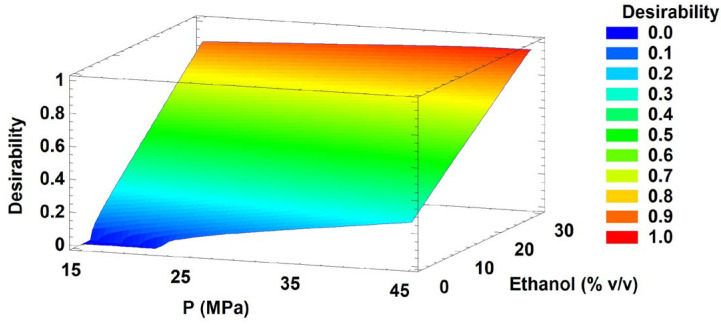
Response surface of the overall desirability as a function of pressure and ethanol concentration at the optimal temperature (60 °C). Abbreviations: pressure (P).

Following this approach, multiple response optimisation identified a global optimum with an overall desirability value of 0.964. The optimal extraction conditions corresponded to the highest levels of the studied factors: 60 °C, 45 MPa, and 30% *v*/*v* ethanol as cosolvent. Under these conditions, the predicted responses were an extraction yield of 35.88%, a TPC of 27.51 mg GAE/g extract, and a TEAC of 2.71 mmol TE/g extract from MTB (Figure 8).

Desirability-based optimisation indicates that the simultaneous maximisation of extraction yield, phenolic content, and antioxidant capacity is favoured at high temperatures and ethanol concentrations, while pressure plays a secondary role within the studied range. The high desirability value obtained reflects a strong compromise among the selected responses, underscoring the effectiveness of ethanol-modified supercritical CO_2_, enhancing the recovery of bioactive compounds. Overall, these findings support the relevance of solvent polarity modulation and thermal effects in driving phenolic extraction and antioxidant potential under optimised supercritical fluid extraction conditions.

### 3.4. Fatty Acids Profile and Other Metabolites

Regarding the traditional folk and medical uses of *Parastrephia* spp., most studies have focused on their anti-inflammatory, gastroprotective, antioxidant, and antibiotic properties [3,26,29]. The Asteraceae family is also a source of FAs, which play a key role in health maintenance. GC–MS analysis was conducted to identify FAs and other relevant compounds in the transesterified fractions obtained from tola. This analysis was performed using both direct biomass and supercritical extracts. The identified compounds, including FAs and other biomolecules, are summarised in Table 4, showing similar extraction profiles across all extracts. The detected masses were compared with reference spectra from established spectral libraries using NIST MS 2.3, enabling tentative identification of compounds based on reported bioactive properties in the literature. As commonly reported in preliminary metabolomic studies, the identification of non-fatty-acid metabolites by GC–MS in this work is exploratory, and the associated biological activities are discussed speculatively and would require validation through targeted analyses in future studies.

Omega-3 and omega-6 FAs, such as linolenic (C18:3) and linoleic (C18:2) acids, were identified in tola extracts. This ratio is very important in diets because these polyunsaturated fatty acids (PUFAs) have opposing effects on metabolic functions in the body [50]. Particularly, omega-3 PUFAs are known to help reduce inflammation and support functions related to cancer, cardiovascular diseases, and metabolic conditions such as diabetes, obesity, osteoporosis, neurological degeneration, and bone fractures [50,51]. Conversely, omega-6 PUFAs are linked to inflammatory processes that increase the risk of atherosclerosis, cardiovascular diseases, and cancer [50,52] if the ratio omega-6/omega-3 is far from the suitable equilibrium. The idea is to find an optimal omega 3/6 ratio for consumers. On the other hand, oleic acid is a type of omega-9 fatty acid and is defined as a monounsaturated fatty acid (MUFA) because it has a double bond at the ninth carbon atom from the methyl end (CH3) of the carbon chain and is broadly known for improving the serum lipoprotein profile (HDL-to-LDL ratio), reducing blood pressure, insulin resistance, and generating positive effects against cardiovascular-risks and obesity [53,54]. The antibacterial activity of unsaturated fatty acids, such as oleic and linoleic acids, has also been demonstrated in the Asteraceae family, mainly against Gram-positive bacteria [55]. The presence of short-chain fatty acids (SCFAs), such as caprylic acid, levulinic acid, methyl isododecanoate (iso-12:0), and methyl 10-methyldodecanoate, represents a promising alternative to conventional harmful solvents. These compounds exhibit advantageous properties, including non-toxicity, high lubricity, thermal stability (e.g., high flash point), and improved flow characteristics at low-temperature conditions [56].

**Table 4:** Fatty acid profile and tentative molecules of essential oils found in the transesterified fractions of tola biomass.

Tentative Compound	Compound Nature	Molecular Formula	RT (min)	Molecular Ion (*m*/*z*) M^+^	Fragments Profile	Biological Activity	References
*Fatty acids*							
Octanoic acid	Caprylic acid	C_9_H_18_O_2_	6.24	158	31, 43, 55, 69, 74, 87, 101, 127, 143, 158	antimicrobial, antioxidant (skin products), treatment of allergy and epilepsy	[57,58]
Pentanoic acid	Levulinic acid	C_6_H_10_O_3_	13.41	130	15, 27, 42, 43, 55, 57, 59, 88, 99, 115	antimicrobial, lubricant, flavourings	[56,59,60]
Undecanoic acid	Methyl isododecanoate (iso-12:0)	C_13_H_26_O_2_	15.06	214	55, 57, 59, 69, 74, 75, 83, 87, 143, 171	cerebrovascular protection	[61]
Dodecanoic acid	Methyl 10-methyldodecanoate	C_14_H_28_O_2_;	17.97	228	55, 57, 69, 74, 83, 87, 97, 143, 185, 199	cerebrovascular protection	[61]
Hexadecanoic acid	Palmitic acid, C_16:0_	C_17_H_34_O_2_	26.88	270	29, 41, 43, 55, 69, 74, 75, 87, 129, 143	antimicrobial, antioxidant	[62]
Methyl stearate	Stearic acid, C_18:0_	C_19_H_38_O_2_	32.42	298	41, 43, 55, 57, 69, 74, 75, 87, 143, 255	hypocholesterolemic, lubricant	[63]
9-Octadecenoic acid	Oleic acid, C_18:1_, ω9	C_19_H_36_O_2_	33.30	296	41, 43, 55, 69, 74, 83, 84, 87, 96, 97	anti-cardiovascular diseases, anti-inflammatory, antioxidant, antiobesity, antibacterial	[55]
9,12-Octadecadienoic acid	Linoleic acid, C_18:2,_ ω6	C_19_H_34_O_2_	35.03	294	41, 55, 67, 68, 79, 81, 82, 95, 96, 109	anti-inflammatory, nematicide, insectifuge, hypocholesterolemic, anticancer, heptaoprotective, antihistaminic, antiacne, antiarthritic, anticoronary, antibacterial	[50,55]
9,12,15-Octadecatrienoic acid	Linolenic acid, C_18:3_ ω3	C_19_H_32_O_2_	37.14	292	41, 55, 67, 79, 80, 81, 91, 93, 95, 108	anti-inflammatory, cancer preventive, anticardiovascular, antimetabolic diseases	[50]
Eicosanoic acid	Arachidic acid, C_20:0_	C_21_H_42_O_2_	37.66	326	41, 43, 55, 57, 69, 74, 75, 87, 143, 326	moderately antioxidant	[64]
Tetracosanoic acid	Lignoceric acid, C_24:0_	C_25_H_50_O_2_	47.26	382	43, 55, 57, 69, 74, 75, 87, 143, 339, 382	antidiabetic	[65]
*Essential oils*							
p-Cymene	monoterpene	C_10_H_14_	4.94	134	39, 41, 65, 77, 91, 115, 117, 119, 120, 134	antioxidant, anti-inflammatory, antinociceptive, anxiolytic, anticancer, antimicrobial	[66,67]
Anisaldehyde dimethyl acetal	phenolic compound	C_10_H_14_O_3_	15.27	182	31, 39, 51, 65, 77, 92, 108, 135, 151, 182	antioxidant, antibacterial, antifungal, antimicrobial, herbicidal	[68]
Anethole	phenylpropanoid	C_10_H_12_O	17.84	148	41, 55, 65, 77, 91, 103, 115, 133, 148	anti-inflammatory, anticarcinogenic and chemopreventive, antidiabetic, immunomodulatory, neuroprotective, antithrombotic	[67,69]
Salvial-4(14)-en-1-one	oxygenated sesquiterpenes	C_15_H_24_O	18.90	220	41, 67, 79, 81, 107, 109, 123, 124, 137, 177	antifungal, antibacterial, antimicrobial, antioxidant, immunological, anti-inflammatory, anti-malarial	[68]
Methyleugenol	analogue of eugenol benzene derivatives	C_11_H_14_O_2_	24.73	178	91, 103, 107, 115, 135, 147, 151, 163, 478, 179	insecticidal, antifeedant (to the larvae)	[70]
2-Propenoic acid, 3-phenyl methyl ester	Cinnamic acid phenolic acids	C_10_H_10_O_2_	26.35	162	51, 77, 102, 103, 104, 131, 132, 161, 162, 163	antioxidant, antimicrobial, anti-inflammatory	[71,72]
Desmethoxyencecalin	benzopyrans (volatile compound)	C_13_H_14_O_2_	38.73	202	43, 63, 86, 115, 116, 144, 145, 187, 188, 202	antioxidant, anti-inflammatory, anticancer, neuroprotective (Alzheimer’s disease)	[73]
2-Propenoic acid, 3-(4-methoxyphenyl)	Ferulic acid (Phenolic acid)	C_11_H_12_O_4_	42.11	192	89, 90, 118, 132, 133, 134, 161, 162, 192, 193	antioxidant, anti-inflammatory	[72]
Atovaquone	naphthoquinone	C_22_H_19_ClO_3_	49.66	366	77, 105, 115, 116, 125, 128, 202, 366, 367, 368	antimicrobial, antipneumocystis (antimalarial capacity)	[74,75]

Additionally, SCFAs serve as effective additives for gasoline and diesel fuels [59,60]. They can also be used as flavouring agents in the fragrance industry [64] or as bioactive compounds with potential health benefits, including Alzheimer’s disease [58], cardiovascular protection [61] and therapeutic roles in allergy and epilepsy treatment [57]. Table 4 also reports the presence of saturated fatty acids (SFAs) from tola extracts. Isomystyric, palmitic, stearic, arachidic and lignoceric acids are the most highlighted in the fatty acids profile. Although the intake of SFAs is associated with an increased cardiovascular risk, they also offer other relevant properties, such as antidiabetic [65], antimicrobial [62], and lubricant [63] properties, which are useful in the biotechnological industry.

Other molecules with biotechnological interest are identified in the fatty acid extracts of MTB. Examples include *p*-cymene, a monoterpene found in over 100 plant species, including members of the Asteraceae [76], which has been reported to display a broad range of biological activities, including antioxidant, anti-inflammatory, antinociceptive, anxiolytic, anticancer and antimicrobial effects [66,67]. Based on the above referred bioactivities, *p*-cymene is considered the most relevant monoterpene compound in aromatic plants. Table 4 also shows the presence of compounds such as anethole, anisaldehyde dimethyl acetal, salvial-4(14)-en-1-one, methyleugenol, 2-propenoic acid, 3-phenyl-, methyl ester, desmethoxyencecalin or 2-propenoic acid, 3-(4-methoxyphenyl)-, methyl ester. They are organic compounds used frequently in the cosmetic and food industries as flavouring agents. Various studies have reported multiple beneficial effects, such as anti-inflammatory, anticarcinogenic and chemopreventive, antidiabetic, immunomodulatory, neuroprotective (for Alzheimer’s disease), antithrombotic, antimicrobial, and insecticidal activities [6,68,69,70,71,72,73]. Notably, the presence of atovaquone confers antimicrobial and antipneumocystis activity on the tola plant. For some time now, scientific studies have investigated the potential use of this compound as an antimalarial drug [74,75]. The identification of these bioactive molecules with therapeutic potential in tola biomass extracts explains the historical use of tola for human health applications from ancient times to the present day.

Table 5 shows the variability of FAs in supercritical extracts of tola, obtained using the factorial design selected for this study. We have highlighted the omega-3 and omega-6 (linolenic and linoleic acids, respectively) as essential FAs, which play a significant role in human and animal metabolism. These FAs have the potential to be elongated and desaturated into arachidonic and eicosapentaenoic acids (EPA, 20:5 ω-3) and docosahexaenoic acid (DHA, 22:6 ω-3). We observed an increase of up to 20% (area) in these FAs under apolar conditions (supercritical carbon dioxide, runs 2–5). Passos et al. [77] also found that increasing pressure and decreasing temperature led to a greater extraction of these compounds from grape seeds, consistent with our findings. Conversely, Follegatti Romero et al. [78] extracted lipids using SFE under various temperature and pressure conditions and found that the highest yield was obtained at 40 MPa and 60 °C. Its highest linolenic acid concentration was achieved under conditions like those in our study. In another study, Pradhan et al. [79] performed extraction on flaxseed. They showed that performing SFE with CO_2_ as the solvent yielded higher concentrations of FA ω-3 and ω-6 than conventional pressure-based extraction. This finding aligns with our results, as the highest concentrations of all fatty acids identified in our experiment, including ω-3 and ω-6 FAs, were obtained using only CO_2_ as the solvent. These results corroborated the relevance of *Parastrephia* sp. as a source of FA of interest and demonstrated that the SFE technique with CO_2_ as the solvent is effective. In the SFA profile, the contents of palmitic (C16:0) and eicosanoid acids (C:20:0) were very high. This indicated that *Parastrephia* sp. can adapt to harsh environmental conditions, since SFAs are associated with a waxy cuticle that protects the shrub from extreme temperature changes during the day, water scarcity, weather fluctuations, and predation [80].

Beyond process optimisation, this study provides insight into how green extraction parameters modulate the chemical and functional profiles of extracts obtained from an ancestrally used medicinal plant. Rather than focusing on the intrinsic superiority of a single extraction technology, the results illustrate the capacity of ethanol-modified supercritical CO_2_ to selectively enrich antioxidant-related fractions with different polarities. This approach contributes to a better understanding of how sustainable extraction strategies can be tailored to valorise traditional plant resources and guide their potential biotechnological or nutraceutical applications.

## 4. Conclusions

The chemical characterisation of native plant species used in Chilean traditional medicine, such as tola herb (*Parastrephia quadrangularis*), contributes to a better understanding of their bioactive composition and supports their scientific documentation. In this study, the total phenolic content, antioxidant activity (TEAC), and fatty acid profile of tola extracts were systematically evaluated using both conventional solvent extraction and ethanol-modified supercritical fluid extraction. In addition, gas chromatography–mass spectrometry analysis enabled the tentative identification of other metabolites of interest, providing an extended chemical profile of the species.

The comparative evaluation of extraction methods, combined with factorial experimental design and kinetic modelling, demonstrated that supercritical fluid extraction allows modulation of extract composition depending on the extraction conditions. Ethanol addition significantly enhanced the recovery of phenolic compounds and affected the extracts’ antioxidant capacity, highlighting the versatility of supercritical fluid extraction as a green extraction strategy for this plant matrix.

Although no direct biological assays were performed to confirm therapeutic effects, the chemical profiles obtained are consistent with previously reported bioactivities associated with *Parastrephia* spp. These findings should, therefore, be interpreted as a basis for future studies to validate specific functional and therapeutic applications and to assess their relevance to food, nutraceutical, or pharmaceutical development under controlled conditions. Future research should focus on compound-specific validation and targeted bioassays to confirm the functional relevance of the identified metabolites, along with comparative evaluations involving other green extraction technologies.

## Figures and Tables

**Table 1:** Factorial design 2^3^, including two centre points, and the results obtained for the Yield, TPC, and TEAC of the tola biomass under different conditions of temperature, pressure, and percentage of ethanol by SFE.

Run	T (°C)	P (MPa)	% Ethanol	Yield (% *w*/*w*)	TPC (mg GAE/g)	TEAC (mmol TE/g)
**1 (cp)**	45	30	15	24.68 ± 0.96	19.66 ± 0.78	2.25 ± 0.09
**2**	30	15	0	5.77 ± 0.23	3.33 ± 0.14	0.02 ± 6.3 × 10^−4^
**3**	60	15	0	5.85 ± 0.23	3.56 ± 0.14	0.02 ± 7.7 × 10^−4^
**4**	30	45	0	5.26 ± 0.21	4.21 ± 0.17	0.02 ± 6.8 × 10^−4^
**5**	60	45	0	9.78 ± 0.38	15.12 ± 0.61	0.09 ± 3.5 × 10^−4^
**6**	30	15	30	33.40 ± 1.32	20.79 ± 0.82	2.98 ± 0.11
**7**	60	15	30	28.94 ± 1.13	27.90 ± 1.10	2.14 ± 0.09
**8**	30	45	30	33.71 ± 1.31	24.81 ± 0.98	0.63 ± 0.02
**9**	60	45	30	34.95 ± 1.37	25.08 ± 0.99	2.91 ± 0.11
**10 (cp)**	45	30	15	25.60 ± 0.99	13.97 ± 0.55	1.78 ± 0.07

Abbreviations: T: Temperature, P: pressure; Yield: extraction yield; TPC: total phenol content; TEAC: Trolox equivalent antioxidant capacity; cp: central point. GAE: gallic acid equivalent; TE: Trolox equivalent. Data are presented as the mean ± standard deviation (SD) of three independent replicates (n = 3).

**Table 2:** The effects of the extracting solvent on the Yield, TPC, and TEAC activity of the tola biomass, determined by conventional extraction.

Solvent	Yield (%, *w*/*w*)	TPC (mg GAE/g)	TEAC (mmol TE/g)
**Water**	24.57 ± 0.58 ^b^	8.05 ± 0.08 ^b^	2.54 ± 0.23 ^a^
**EtOH**	31.73 ± 0.26 ^a^	21.74 ± 0.20 ^a^	2.39 ± 0.09 ^a^

Abbreviations: EtOH: Ethanol; Yield: Extraction yield; TPC: total phenol content; GAE: gallic acid equivalent; TEAC: Trolox equivalents antioxidant capacity; TE: Trolox equivalent. The results are averaged (n = 3; ±SD), and the different letters (a and b) indicate that these pairs show statistically significant differences at the 95.0% confidence level.

**Table 3:** Adjusted kinetic parameters of the spline linear model to supercritical fluid extraction (SFE) from the tola biomass; P = 30 MPa, T = 45 °C, and CO_2_ + ethanol (85:15 *v*/*v*) flow rate.

Parameters		Stages of the OEC		
		CER	FER	DC
Time (min)		16.78	31.42	180.0
Accumulated extract at each stage (%)		20.01	3.79	5.17
Accumulated extract total (%)		20.01	23.80	28.97
Recovery (%)		69.05	13.10	17.85
Total recovery (%)		69.05	82.15	100.0
M_ext_ (g extract/min)		2.4 × 10^−2^	5.45 × 10^−3^	6.82 × 10^−4^
Y (mg extract/g biomass)		199.94	49.42	66.07
Y* (g extract/g _(CO2 85% + Ethanol 25%)_)		7.3 × 10^−3^	1.7 × 10^−3^	2.1 × 10^−4^
R^2^		0.9976	0.9987	0.9993
**Linear coefficients of the spline model**	***b*_0_**	***a*_1_**	***a*_2_**	***a*_3_**
	−1.4333	1.306	−1.059	−0.2177

$b_{0}$: Linear coefficient of the first line (CER); $a_{1},a_{2}\;and\;a_{3}:$ the intercepts of lines 1, 2, and 3 corresponding to the periods CER, FER, and DC, respectively; $t_{CER}\;and\;t_{FER}:$ Times in the intercepts of lines 1 and 2, and lines 2 and 3, respectively; $M_{EXT}\left(t\right):$ mass of the extract at time t; $Y_{t}$: Variable response for the consideration stage (CER, FER, and DC); Y*: Variable response for the consideration stage (CER, FER, and DC).

**Table 5:** The fatty acid profile (% area *) present in the tola biomass under a factorial design 2^3^ with two centre points using SFE.

Run	1 (cp)	2	3	4	5	6	7	8	9	10 (cp)
**T (°C)**	45	30	60	30	60	30	60	30	60	45
**P (MPa)**	30	15	15	45	45	15	15	45	45	30
**% Ethanol**	15	0	0	0	0	30	30	30	30	15
**SCFAs**	23.42	23.49	27.07	14.60	13.47	14.01	14.82	17.94	15.63	21.56
**SFA**	58.44	50.79	45.90	55.14	60.42	58.09	63.98	55.04	55.64	66.24
**UFA**	18.14	25.72	27.02	30.26	26.11	27.90	21.21	27.02	28.72	16.20
**ω-3 + ω-6**	9.63	19.43	20.24	20.59	18.06	19.26	12.50	19.15	18.27	8.02
**ω-9**	8.51	6.29	6.78	9.67	8.05	8.64	8.71	7.87	10.45	8.18

Abbreviations: temperature (T), pressure (P); central point (cp); short-chain fatty acids (SCFAs); saturated fatty acid (SFA); unsaturated fatty acid (UFA); ω-3 + ω-6 = Linolenic and linoleic acids; ω-9: oleic acid. * The abundance of each fatty acid was expressed as its relative percentage, calculated as the ratio of its individual peak area with respect to the integration area of all peaks obtained by GC-FID, in percentage (*p* < 0.05; n = 3; SD ≤ 5%).

## Data Availability

The original contributions presented in this study are included in the article/Supplementary Materials. Further inquiries can be directed to the corresponding authors.

## Supplementary Materials

The following supporting information can be downloaded at https://www.mdpi.com/article/10.3390/antiox15030303/s1. Table S1: Regression coefficients and *p*-Value for Yield, TPC, and TEAC in their original units and statistics for the fit obtained by multiple linear regression using SFE.

## Abbreviations

The following abbreviations are used in this manuscript:

CER	Constant Extraction Rate
FA/FAs	Fatty Acid/Fatty Acids
FAMEs	Fatty Acid Methyl Esters
FER	Falling Extraction Rate
GC–MS	gas chromatography–mass Spectrometry
MTB	Milled Tola Biomass
OEC	Overall Extraction Curve
RSM	Response Surface Methodology
SFE	Supercritical Fluid Extraction
TPC	Total Phenolic Content
TEAC	Trolox Equivalent Antioxidant Capacity
