# Supplementary material for: Improvement in the Extraction of Antioxidant-Related Compounds from Parastrephia quadrangularis (“tola”) Using Ethanol-Modified Supercritical Carbon Dioxide
Source: Antioxidants (Basel). 2026 Feb 28;15(3):303. doi: 10.3390/antiox15030303 (PMC13024668; doi:10.3390/antiox15030303)
Supplement: Supplementary file 1 [file antioxidants-15-00303-s001.zip › antioxidants-4155531-supplementary.pdf]

Supplementary material

Table S1. Regression coefficients and p-Value for Yield, TPC, and TEAC in their original units and statistics for the fit obtained by multiple linear regression using SFE.

**Terms of the model**	**Yield**		**TPC**		**TEAC**	
	Estimate	***p-value***	***Estimate***	***p-value***	***Estimate***	***p-value***
constant	10.1965		10.603		2.52525	
A: T (°C)	-0.0923333	0.9105	-0.211667	0.0325*	-0.052	0.6071
B:P (MPa)	-0.1965	0.4519	-0.388667	0.5772	-0.0785833	0.6071
C: Ethanol (% v/v)	1.01667	0.0027*	0.551	0.0143*	0.0641667	0.0484*
AB	0.00563333	0.4355	0.0132444	0.1015	0.00177222	0.3131
AC	-0.00434444	0.5385	0.00902222	0.2097	0.000761111	0.6394
BC			-0.0173556	0.0549	-0.000916667	0.5760
**Statistics for the goodness of fit of the model**						
R^2	0.966831			0.951096		0.814672
adjusted R^2	0.90493			0.853287		0.444016
RSD	3.99337			3.60734		0.932589
P	0.2403			0.2510		0.7482

Note: R^2–determination coefficient, adjusted R^2, RSD–residual standard deviation, P-value of the lack-of-fit test for the model; * - significant coefficients of the model, T–Temperature (ºC), P – pressure (MPa) and Ethanol – percentage of cosolvent (%v/v).
